# Evaluation of Impact of a Pharmacist-Led Educational Campaign on Disease Knowledge, Practices and Medication Adherence for Type-2 Diabetic Patients: A Prospective Pre- and Post-Analysis

**DOI:** 10.3390/ijerph191610060

**Published:** 2022-08-15

**Authors:** Yusra Habib Khan, Abdulaziz Ibrahim Alzarea, Nasser Hadal Alotaibi, Ahmed D. Alatawi, Aisha Khokhar, Abdullah Salah Alanazi, Muhammad Hammad Butt, Asrar A. Alshehri, Sameer Alshehri, Yasser Alatawi, Tauqeer Hussain Mallhi

**Affiliations:** 1 Department of Clinical Pharmacy, College of Pharmacy, Jouf University, Sakaka 72388, Saudi Arabia; 2 Health Sciences Research Unit, Jouf University, Sakaka 72388, Saudi Arabia; 3 Institute of Pharmacy, Lahore College for Women University, Lahore 54000, Pakistan; 4 Faculty of Pharmacy, University of Central Punjab, Lahore 54000, Pakistan; 5 Infection Control Department, Alameen Hospital, Taif 26511, Saudi Arabia; 6 Department of Pharmaceutics and Industrial Pharmacy, College of Pharmacy, Taif University, Taif 21944, Saudi Arabia; 7 Department of Pharmacy Practice, Faculty of Pharmacy, University of Tabuk, Tabuk 71491, Saudi Arabia

**Keywords:** T2DM, pharmacist, intervention, COVID-19, knowledge, practices, medication adherence, pre-post analysis

## Abstract

Type 2 Diabetes mellitus is a major public health concern with an alarming global growth rate. According to the World Health Organization (WHO), Saudi Arabia ranks seventh in the world and second in the Middle East for the largest estimated burden of diabetic cases. Evidence shows that pharmacist-led care programs can be beneficial for the effective treatment of diabetes mellitus. Current study was aimed to evaluate the impact of Pharmacist-Based Diabetic Intervention (PDIM) for Type 2 Diabetes patients on knowledge of the disease, adherence to medications and self-care practices during the first wave of COVID-19. A multi-arm pre-post study was conducted among type 2 diabetic patients from April to October 2021 in Sakaka, Saudi Arabia. Patients were randomly divided into an intervention and a control group. The intervention group received the PDIM, whereas the control group only received the usual care. The pharmacist-based diabetes intervention model consisted of a diabetic educational module and medication improvement strategies. Furthermore, the intervention group also received specific telepharmacy services (calls, messages or emails) to address their medication-related problems, inquire about medication adherence and follow-up. At the end of six months, disease knowledge, self-care practices, and medication adherence score were analyzed. Furthermore, HbA1c and lipid profile were also compared. A total of 109 patients were included in the study. A significant difference was observed in the knowledge score between the intervention and control group (16.89 ± 2.01 versus 15.24 ± 2.03, *p*-value < 0.001). Similarly, self-care practices also improved in the intervention group as compared to the control group (4.39 ± 1.10 versus 3.16 ± 0.97, *p*-value < 0.001). Furthermore, the medication adherence and HbA1c significantly improved during between the group analysis (*p* < 0.05). Our study demonstrates that pharmacist-based diabetes intervention model is effective in improving patients’ knowledge of diabetes, self-care practices, medication adherence and glycemic control.

## 1. Introduction

Type 2 diabetes mellitus (DM2) is the most prevalent type of diabetes, accounting for 85–95% of all diabetes cases. Approximately 4.15 million people are affected by DM2 globally, with the number expected to increase to 592 million by 2035. According to the latest World Health Organization (WHO) figures, Saudi Arabia ranks seventh in the world and second in the Middle East for the largest estimated burden of diabetic cases [1]. Moreover, the staggering increase in T2DM cases eventually leads to premature mortality and morbidity due to several microvascular complications. However, effective maintenance of glycemic control can play a vital role. It will not only be beneficial in improving the quality of life and survival rate of T2DM patients but also reduce the burden on the healthcare system. Despite advances in drug therapy and the management of diabetes, glycemic control remains a challenge without adherence to medications. Non-adherence to therapy and sedentary lifestyle is the major obstacles in the management of T2DM [2,3,4]. Therefore, the development of innovative strategies to enhance patient care and medication adherence in diabetes is of paramount importance.

An integrated approach involving mediations, lifestyle modifications, and strict diet is important for the effective treatment of DM2. Pharmacist-based interventional studies have significantly improved clinical outcomes among diabetic patients and have underlined the importance of pharmacists in glycemic control and medication adherence [5,6]. However, these pharmacist-based clinical services were greatly affected during the catastrophic waves of COVID-19. A large percentage of patients with chronic disease did not attend their follow-up visits at the hospital. Furthermore, during the COVID-19 pandemic, routine care of diabetic patients visiting hospitals for both follow-up or management of complications changed due to repeated lockdowns, overburdened hospitals, cancelation of hospital appointments and patients’ reluctance to visit hospitals due to the fear of contracting infection at the hospital [7,8,9,10].

Therefore, a Pharmacist-based Diabetes Intervention Model (PDIM) was designed to improve diabetes care in the Saudi population with Type 2 diabetes during the first wave of the COVID-19 pandemic. The primary aim of the current study was to evaluate the impact of Pharmacist-based Diabetic Intervention (PDIM) for Type 2 Diabetes patients on disease knowledge, medication adherence and self-care practices. Furthermore, the secondary objective was to examine the effect of PDIM on the physiological profile of Type 2 Diabetes patients.

## 2. Materials and Methods

### 2.1. Ethical Statement

The study was approved by the Local Committee on Bioethics (LCBE) at Jouf University, Saudi Arabia (Ref: 05-08-42). Informed consent was obtained from each participant prior to the start of the study. The identity of each participant was kept confidential throughout the analysis.

### 2.2. Study Design and Setting

A multi-arm pre-post prospective study was conducted among type 2 diabetic patients visiting community pharmacies in Sakaka, Saudi Arabia. The follow-up period for each study participant was six months, i.e., from April 2021 to October 2021.

### 2.3. Sample Size Calculation

The study sample size was calculated based on the effect size of glycated hemoglobin (HbA1C) reduction as 0.7% [11]. The formula given above was used to calculate the study sample size keeping *a* = 1.96, *b* = 1.28, *σ* = 0.7, *μ*_1_ − *μ*_2_ = 0.5. Since it was a multi-arm study, a sample size of 42 patients per group was obtained. However, considering the attrition rate of 25%, a total of 110 study participants was considered sufficient for the current study.

$$N\;\left(sample\;size\right)=\frac{2\;\left[{\left(a+b\right)}^{2}\sigma^{2}\right]\;}{{\left(\mu_{1}-\mu_{2}\right)}^{2}}$$

### 2.4. Inclusion/Exclusion Criteria

Patients visiting a community pharmacy with a confirmed diagnosis of poorly controlled type 2 DM (HbA1C > 7%), age > 30 years were included in the study. Patients having serious renal or hepatic dysfunction, and pregnant females were excluded from the study owing to altered physiological functions. In addition, patients with hearing or vision impairments and psychological problems were also excluded from the current study. Lastly, patients diagnosed with Type 1 DM and gestational diabetes were also excluded from the study. Any patient having missing HbA1C values or having a last recorded value of greater than 6 months were excluded.

### 2.5. Data Collection

All patients who agreed to participate were enrolled in this study. Patients were stratified into an intervention group (IG) and a control group (CG) through the block randomization method. The IG underwent PDIM while the CG merely received the usual care. Patient objective data were collected through medical records and direct interviews. The baseline HbA1C data and lipid profile values were recorded from patient follow up records.

### 2.6. Study Tool

**Figure 1:**
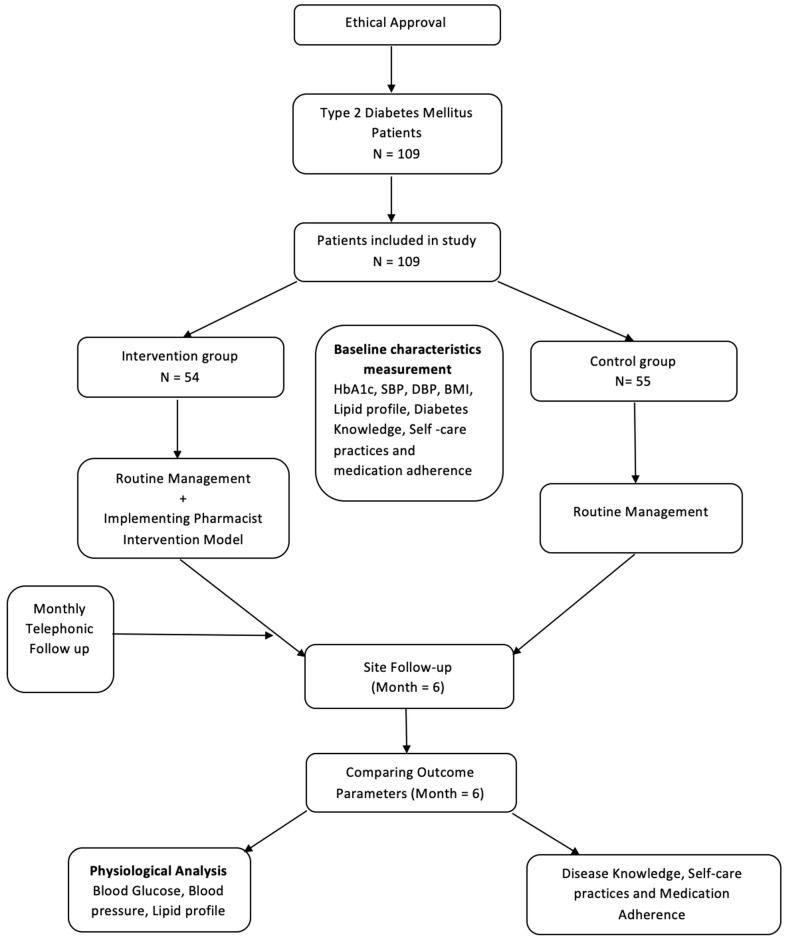
Study flow diagram.

The study tool was developed after an extensive review of the literature. The study instrument comprised different sections:

(1) ***Diabetes Knowledge Assessment:*** Following sample collection, all patients were asked to complete a self-constructed and validated questionnaire on diabetes knowledge and self-practice. Each correct answer scored 1, otherwise zero. The diabetes knowledge score ranged from 0 to 20. However, self-care practices score for T2DM ranged from 0 to 4.
(2) ***Medication Adherence:*** The medication adherence was assessed by using the 6-item modified Morisky scale (MMS) with different questions. The adherence score ranged from 0 to 6, where a higher score correlated with higher adherence.
(3) ***Pharmacist Intervention Model:*** Following the initial stratification and assessment, the IG underwent PDIM which included a diabetic educational module and medication improvement strategies. The diabetic educational module consisted of a pharmacist-led informative session with a primary focus on knowledge about diabetes and medication adherence. A diabetes self-care brochure and information material were provided to the patients in IG. In addition, an interactive session was organized that focuses on the causes of diabetes, risk factors associated with disease, awareness of uncontrolled diabetes, strategies to control disease, and recommendations on healthy food items for effective management of diabetes. Medication improvement strategies included counseling on the importance of medication adherence in the control of diabetes.
(4) ***Tele-pharmacy Services:*** Considering the movement restrictions due to the COVID-19 pandemic, the pharmacist also provided telepharmacy services (calls, messages, or emails) to patients in order to cater their medication-related problems, inquire about medication adherence and follow-up. Telepharmacy services also included pictorial messages to patients that focused primarily on the effective use of diabetes medicine, maintaining a predefined glycemic control and eating habits.
(5) ***Post Intervention Follow up:*** After baseline measurements and the implementation of PIDM, the IG received telepharmacy services every month for 6 months, while the control group was not contacted. The primary and secondary study outcomes were measured in both groups at the end of the study. The study methodology is briefly explained in the flow chart Figure 1.

### 2.7. Outcome Measured

The primary study outcome was the evaluation of disease knowledge and self-care practices, and medication adherence. However, the secondary study outcomes were assessment of HbA1C, blood pressure, and Lipid Profile (LDL, HDL and TGs).

### 2.8. Data Analysis

Data were analyzed using SPSS, IBM (Chicago, IL, USA) version 22.0. All continuous data were presented as mean with standard deviation, counts with proportion and median with interval, where appropriate. Descriptive and inferential statistics were applied after fulfilling the analysis assumption. The continuous variables were analyzed through Independent *t*-test, whereas the categorical variables were analyzed using the chi-square test. Mean difference among scores and effect size was calculated by applying a paired sample *t*-test. A *p*-value less than 0.05 was considered significant throughout the analysis.

## 3. Results

### 3.1. Characteristics of Study Participants

A total of 109 T2DM patients, 55 in the control group and 54 in the interventional group, completed this study. The average age of the study participants was 58.33 ± 7.68 years. Approximately 45.9% of the patients had a smoking history. Almost a third (36.7%) of the study population had a family history of diabetes. The majority of patients (86.2%) were on more than one oral antidiabetic medication to maintain the optimum blood glucose level and to avoid any complication. About 78% of the patients had comorbidities along with T2DM. The detailed demographic characteristics of each group are explained in Table 1.

### 3.2. Knowledge, Practices and Medication Adherence

**Figure 2:**
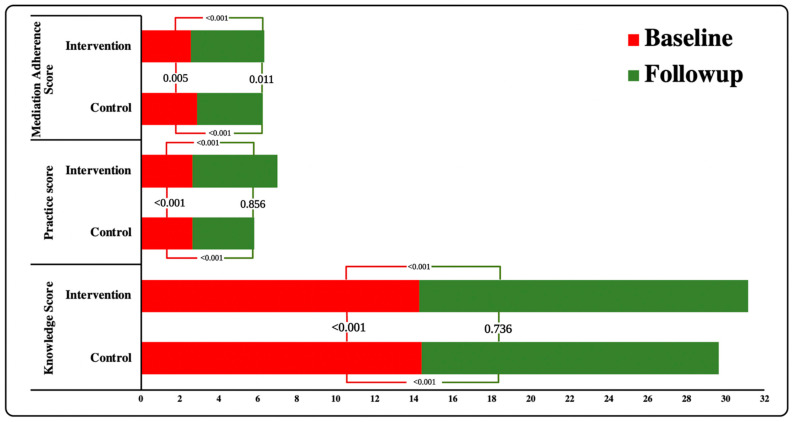
Association of Knowledge, Practices, and Medication Adherence Scores within and between the groups.

Knowledge, self-care practice, and medication adherence showed pronounced improvements during follow-up (Table 2). A significant difference was observed in the knowledge and self-care practices score of IG and CG as well as baseline and follow-up (Figure 2). Similarly, the IG medication adherence score was much higher in follow-up compared to the CG (*p* = 0.005). During the analysis between groups, a significant improvement in IG was observed compared to CG (*p* < 0.001).

### 3.3. Physiological Characteristics

**Figure 3:**
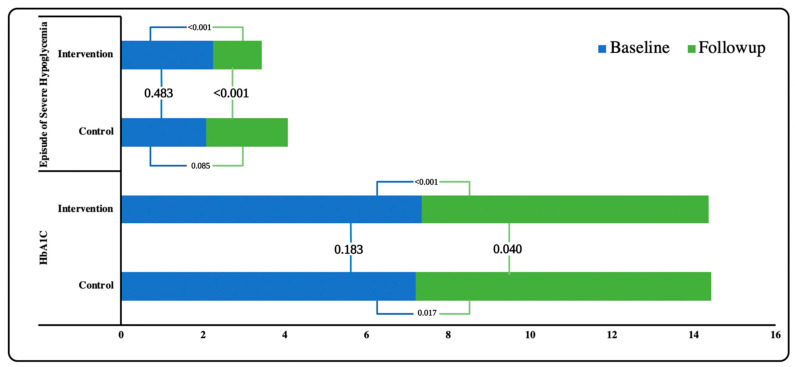
Association of Blood glucose parameters within and between the groups.

**Figure 4:**
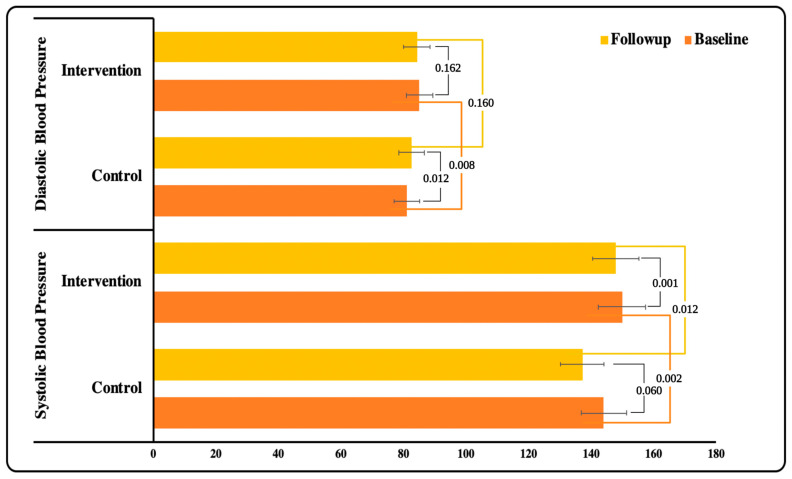
Association of Blood pressure within and between the groups.

**Figure 5:**
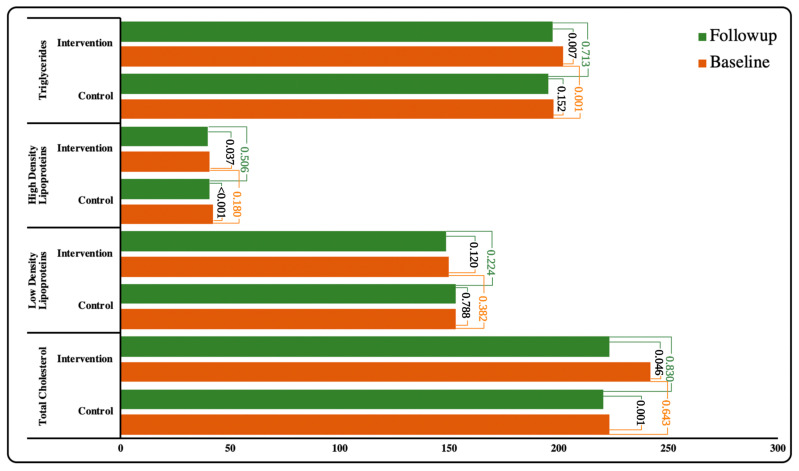
Association of Lipids profile within and between the groups.

The physiological characteristics of the study participants at baseline and follow-up between the control and intervention groups were shown in Table 3. A significant difference was observed in the HbA1c level in IG compared to CG (*p* = 0.040). Furthermore, during the ‘intergroup analysis’, a significant difference was observed in hypoglycemia episodes at follow-up between IG and CG (*p* < 0.001) (Figure 3). Similarly, blood pressure (SBP and DBP) in both groups improved. However, the difference in DBP between the groups was not statistically significant (Figure 4). The lipid profile of both the groups improved upon follow-up but no significant variation was observed between the groups. However, during ‘within the group analysis’, a significant difference was observed in total cholesterol, HDL and triglycerides in IG upon follow up (*p* < 0.05) (Figure 5).

## 4. Discussion

To our knowledge, this is the first study to design and implement the pharmacist-based diabetes intervention model for Type 2 diabetes in Saudi Arabia. The effectiveness of PDIM was evident through improvements in knowledge, medication adherence, self-care practices and physiological characteristics. Our findings illustrate that males outnumbered females when visiting community pharmacies. Similar dominance was also reported in previous studies [11,12]. Smoking and obesity are the two main risk factors associated with diabetes mellitus [13,14]. Most of the diabetic patients had a history of smoking in our study. Consistent with the previous study, the majority of our study population had had diabetes for ≥5 years [15].

A considerable improvement in the knowledge score was observed in the IG as compared to the CG after the implementation of PDIM. Our findings indicate that individualized and comprehensive counseling sessions by Pharmacists can be beneficial to enhance the patient’s knowledge regarding their disease. Similar results were also reported by a study in India, with a significant rise in knowledge scores following pharmacist-led T2DM counseling programs [10,16,17].

There is a strong relationship between patient knowledge of the disease and adherence to medication. It is hypothesized that a well-informed patient has a better understanding of their disease and treatment process [18]. Poor adherence is an major obstacle to achieving the desired therapeutic goals in DM [19]. It is observed that poor medication adherence is associated with poor glycemic control in T2DM patients [20]. Considering our results, the medication adherence improved in both the groups upon follow-up, but the score was significantly higher in the IG. This might be due to the pharmacist’s involvement and telephonic follow-up that addressed patient medication-related problems and emphasized medication adherence. These findings are consistent with those of a recent study that found increased medication adherence in T2DM following six months of pharmacist assistance [21,22,23].

The management of DM, in addition to the optimum glycemic control includes effective strategies to limit the associated disability and mortality in patients [24]. The American Diabetes Association (ADA) has created a list of critical self-care practices that can be used to keep blood glucose levels at acceptable levels, minimize diabetes complications, and improve diabetic patients’ quality of life. Blood glucose monitoring, healthy eating habits, physical activity, and diabetic foot care are all examples of diabetes self-care [25]. In our study, self-care activities also improved in the IG after the implementation of PDIM. Previous studies also reported similar significant improvements in self-care practices after the successful pharmacist-led care program [26,27,28].

Similarly, pharmacist-led interventions remarkably improve the HbA1c levels in T2DM [29]. In our study, during physiological analysis, IG showed a significant reduction in HbA1c levels compared to CG. Although little improvement was observed in HbA1c level in IG upon follow up, this positive outcome can be linked to the improved diabetes knowledge and self-care practices in IG. This is consistent with earlier research, which found a 0.5-1.0 percent drop in HbA1c levels [15,30,31]. Furthermore, a considerable reduction in hypoglycemic episodes in the IG suggests that patient education regarding glucose monitoring and hypoglycemia has a favorable impact on management of T2DM.

However, no significant improvement was observed in the lipid profile in the IG group upon follow-up. Although total cholesterol, LDL and TGs levels decreased during the follow-up in the IG, no significant reduction was observed between the IG and the CG. As there were no changes in pharmacotherapy during the follow-up period, we hypothesize that merely good dietary habits could not mediate a change in lipid profile. These findings are in contrast to those of the 12-month research carried out in Northern Cyprus [26]. It can be inferred that perhaps the short length of the follow-up period in our investigation contributed to these findings. A longer follow-up period may be beneficial in determining the influence of PDIM on the physiological profile of Type 2 Diabetes patients.

Therefore, the involvement of pharmacist in diabetes care, both in hospital and in the community pharmacies can be beneficial in improving the glycemic control, adherence, disease-related knowledge, self-practices, drug-related problems and patient satisfaction where diabetes is concerned. Diabetes control clinics led by pharmacists in hospitals and community pharmacies can also be established to help reduce disease progression and mortality.

Despite being the first study to introduce a pharmacist-based diabetes intervention model in Saudi Arabia, there are a few limitations of our study. The smaller sample size and shorter follow-up period limit the applicability of the findings, as diabetic patients require continuous follow-up. However, the present study is strengthened being the first study to initiate diabetes care at community pharmacies through face-to-face interaction and telepharmacy services during the COVID-19 pandemic in Saudi Arabia. Furthermore, the findings may be applicable to other regions of the world in a population of similar patients, as baseline demographics were matched between two groups through appropriate randomization. This research is relatively preliminary and replication is encouraged. Further studies are required to confirm the capacity of such interventions in pharmacy practice.

## 5. Conclusions

Our study demonstrates that the pharmacist-based diabetes intervention model is effective in improving knowledge of the disease, self-care practices and medication adherence in the Type 2 Diabetes mellitus population resulting in better glycemic control and fewer hypoglycemic episodes. It would also help to reduce the increasing burden of diabetes in Saudi Arabia by improving patient adherence and quality of life. Furthermore, telepharmacy services will also increase the opportunities for pharmacists to play a pivotal clinical role in the management of chronic diseases.

## Figures and Tables

**Table 1:** Demographic characteristics of the study participants.

Characteristics	Overall (N = 109)	Control (N = 55)	Intervention (N = 54)	*p*-Value
**Age (mean ± SD)**	58.33 ± 7.68	57.84 ± 7.88	58.83 ± 7.51	0.501
**Age groups**				
36–45	8 (7.3)	3 (5.5)	5 (9.3)	0.572
46–55	30 (27.5)	17 (30.9)	13 (24.1)	
56–65	51 (46,7)	27 (49.1)	24 (44.4)	
>Or = 66	20 (18.3)	8 (14.5)	12 (22.2)	
**Gender**				
Male	65 (59.6)	33 (60.0)	32 (59.3)	0.546
Female	44 (40.4)	22 (40.0)	22 (40.7)	
**BMI**	24.65 ± 2.19	24.29 ± 2.31	25.02 ± 2.02	0.02
**Smoking History**	50 (45.9)	23 (41.8)	27 (50.0)	0.253
**History of Diabetes**	40 (36.7)	22 (40.0)	18 (33.3)	0.301
**Duration of Diabetes (years)**	6.85 ± 2.86	6.42 ± 3.37	7.30 ± 2.17	0.038
**Number of Comorbidities**				
None	13 (11.9)	8 (14.5)	5 (9.3)	0.312
One	13 (11.9)	9 (16.4)	4 (7.4)	
Two	45 (41.3)	18 (32.7)	27 (50.0)	
Three	35 (32.1)	18 (32.7)	17 (31.5)	
Four	3 (2.8)	2 (3.6)	1 (1.9)	
**Antihypertensive**	75 (68.8)	34 (61.8)	41 (75.9)	0.083
**Lipid Lowering Drugs**	79 (72.5)	41 (74.5)	38 (70.4)	0.392
**No. Of Oral Antidiabetics Medications**				
One	15 (13.8)	11 (20.0)	4 (7.4)	0.135
Two	54 (49.5)	22 (40.0)	32 (59.3)	
Three	35 (32.1)	19 (34.5)	16 (29.6)	
Four	5 (4.6)	3 (5.5)	2 (3.7)	

Values are expressed as Frequency (Percentage). *p* < 0.05 is calculated between intervention and control group.

**Table 2:** Comparison of Knowledge, Practices, and Medication Adherence Score at Baseline and Follow-up.

Group	Variables	Baseline	Follow Up	Mean Difference *	Confidence Interval	Effect Size (R)
**Control**	Knowledge Score	14.40 ± 2.131	15.24 ± 2.036	−0.836 ± 0.966	−1.106 to −0.567	0.418
	Practice score	2.65 ± 0.700	3.16 ± 0.977	−0.509 ± 0.742	−0.71 to −0.308	0.324
	Mediation Adherence Score	2.89 ± 0.712	3.35 ± 0.751	−0.455 ± 0.603	−0.618 to −0.292	0.367
**Intervention**	Knowledge Score	14.26 ± 2.216	16.89 ± 2.016	−2.63 ± 1.521	−3.045 to −2.215	0.753
	Practice score	2.63 ± 0.734	4.39 ± 1.106	−1.759 ± 1.317	−2.119 to −1.4	0.645
	Mediation Adherence Score	2.54 ± 0.719	3.78 ± 0.816	−1.241 ± 0.91	−1.489 to −0.992	0.655

Values are expressed as Mean ± SD, Independent sample *t*-test. * Difference in mean score of follow-up from baseline.

**Table 3:** Physiological characteristics of study participants.

Group	Variables	Baseline	Follow Up	Mean Difference *	Confidence Interval	Effect Size (R)
**Control**	HbA1c	7.189 ± 0.668	7.238 ± 0.643	−0.0491 ± 0.1477	−0.089 to −0.0092	0.101
	Episodes of severe hypoglycemia in past 6 months	2.07 ± 1.230	2.00 ± 1.072	0.073 ± 0.766	−0.134 to 0.28	0.009
	Systolic Blood Pressure	144. 07 ± 9.695	137.16 ± 29.64	6.909 ± 26.699	−0.309 to 14.127	0.064
	Diastolic Blood Pressure	81.00 ± 7.191	82.53 ± 6.713	−1.527 ± 4.354	−2.704 to −0.35	0.111
	Total Cholesterol	222.98 ± 31.14	220.36 ± 32.30	2.618 ± 13.365	−0.995 to 6.231	0.038
	Low Density Lipoproteins	153.05 ± 20.66	152.78 ± 19.99	0.273 ± 7.499	−1.755 to 2.3	0.001
	High Density Lipoproteins	42.02 ± 7.269	40.31 ± 6.563	1.709 ± 2.006	1.167 to 2.251	0.425
	Triglycerides	197.60 ± 50.09	195.15 ± 48.53	2.455 ± 5.167	1.058 to 3.851	0.187
**Interventional**	HbA1c	7.352 ± 0.597	7.011 ± 0.4878	0.3407 ± 0.243	0.2744 to 0.4071	0.667
	Episodes of severe hypoglycemia in past 6 months	2.24 ± 1.008	1.20 ± 0.959	1.074 ± 1.043	0.789 to 1.359	0.519
	Systolic Blood Pressure	149.87 ± 9.641	147.96 ± 9.210	1.907 ± 3.901	0.843 to 2.972	0.196
	Diastolic Blood Pressure	85.07 ± 8.565	84.33 ± 6.602	0.741 ± 3.837	−0.307 to 1.788	0.037
	Total Cholesterol	241.72 ± 25.22	223.24 ± 47.90	18.481 ± 47.985	5.384 to 31.579	0.131
	Low Density Lipoproteins	149.85 ± 17.22	148.50 ± 16.32	1.352 ± 6.286	−0.364 to 3.068	0.045
	High Density Lipoproteins	40.28 ± 6.132	39.52 ± 5.75	0.759 ± 2.613	0.046 to 1.472	0.079
	Triglycerides	201.76 ± 43.07	197.02 ± 42.24	4.741 ± 17.03	0.093 to 9.389	0.073

Values are expressed as Mean ± SD, Independent sample *t*-test. * Difference in mean score of follow-up from baseline.

## Data Availability

Not applicable.
